# Effects of Breaking Methods on the Viscosity, Rheological Properties and Nutritional Value of Tomato Paste

**DOI:** 10.3390/foods10102395

**Published:** 2021-10-09

**Authors:** Ruiping Gao, Zhen Wu, Qian Ma, Zhiqiang Lu, Fayin Ye, Guohua Zhao

**Affiliations:** 1 College of Food Science, Southwest University, Chongqing 400715, China; gaorp@ctbu.edu.cn (R.G.); zhenwu1985@163.com (Z.W.); maggie0822@email.swu.edu.cn (Q.M.); lzq0812@163.com (Z.L.); fye@swu.edu.cn (F.Y.); 2 College of Environment and Resources, Chongqing Technology and Business University, Chongqing 400067, China; 3 Chongqing Key Laboratory of Chinese Medicine & Health Science, Chongqing Academy of Chinese Materia Medica, Chongqing 400065, China; 4 Chongqing Engineering Research Center of Regional Foods, Southwest University, Chongqing 400715, China

**Keywords:** tomato paste, breaking methods, ultrasound, pectin-related enzyme, lycopene, large amplitude oscillatory shear (LAOS)

## Abstract

Ultrasound-assisted processing has potential application advantages as an emerging technology for preparing tomato paste. This work explored the influence of ultrasound break at 22 °C (US-Break-22) and 65 °C (US-Break-65) on the viscosity, rheological properties and nutritional values of newly prepared tomato paste, compared with traditional thermal break at 65 °C (Break-65) and 90 °C (Break-90). Results showed that the US-Break-65 paste had the largest apparent viscosity, yield stress, consistency coefficient, solid-like nature, and large amplitude oscillatory shear behavior, followed by the US-Break-22 paste, Break-90 paste, and Break-65 paste. Based on the results of the pectin-related enzymes, particle size, and serum pectin of the pastes, it was revealed that the above-mentioned properties were mainly determined by the particle size and pectin content in their serum. The level of ascorbic acid followed the order of US-Break-22 paste > US-Break-65 paste > Break-65 paste > Break-90 paste. The level of total carotenoids followed the order of US-Break-22 paste ≈ US-Break-65 paste > Break-90 paste ≈ Break-65 paste. The level of total *cis*-carotenoids followed the order of US-Break-65 paste > US-Break-22 paste > Break-90 paste > Break-65 paste. The level of phenolics and antioxidant activities followed the same order of US-Break-22 paste > US-Break-65 paste > Break-90 paste > Break-65 paste. Overall, the viscosity, rheological properties and nutritional values of the tomato pastes prepared by US-Break-65 and US-Break-22 were significantly higher than those prepared by Break-65 and Break-90. Therefore, ultrasound assisted processing can prepare high quality tomato paste and can be widely implemented in the tomato paste processing industry.

## 1. Introduction

Tomato (*Lycopersicon esculentum* Mill.) is one of most popular vegetables and is consumed either fresh or from its derived products, including canned tomatoes, juices, sauces, purees, and pastes [1]. Several studies have confirmed that the tomato and its derived products have excellent antioxidant, anti-inflammatory, and anticancer activities [2]. These health-promoting effects have been widely ascribed to the presence of carotenoids (lycopene, β-carotene, lutein), phenolics and ascorbic acid [3].

As one of the most important derived products, tomato paste plays a vital role in the human diet. Viscosity is one of the most crucial quality parameters of tomato paste, determining the texture and consistency of the products [4]. Several studies have reported that the viscosity of tomato paste is directly related to the activity of pectin methylesterase (PME) and polygalacturonase (PG), which can cause the degradation of cell wall pectin. PME catalyzes the pectin de-esterification, and the PG further catalyzes the hydrolytic cleavage of the α-D-1,4-glycosidic bonds within the pectin acid chain. This causes the depolymerization of cell wall pectin when these two enzymes act synergistically, resulting in a decrease in the viscosity of tomato products [5,6]. In this regard, tomato pastes production is performed by two traditional thermal processing methods, including cold break and hot break. Generally, cold break is performed at a temperature below 70 °C, whereas hot break is performed at temperature between 85 to 102 °C. Cold break only inactivates pectin-related enzymes partially and yields a paste with low viscosity. However, cold break paste has a good color and flavor due to the lower processing temperature. Hot break can inactivate PME and PG completely and yield a tomato paste with high viscosity. Although a high viscosity paste can be obtained by hot break, it simultaneously leads to flavor losses, browned color and nutritional degradation. Cold break paste is suited to further prepare diluted tomato juice and vegetable cocktails with a more natural color and fresher tomato flavor, while hot break paste is suited to produce pizza sauce and ketchup with high viscosity [7,8,9,10]. Therefore, it is clearly found that the tomato paste produced by the conventional heat treatment methods does not have high viscosity, good flavor and nutritional value simultaneously. In this scenario, it is worthwhile to investigate novel methods to prepare high quality tomato pastes with high viscosity, good flavor and nutritional value.

Power ultrasound has been regarded as one of the most promising alternative technologies to replace conventional thermal processing, which causes food quality deterioration. On the one hand, ultrasound is a non-thermal technique that can effectively inactivate enzymes in fruit and vegetable products. It has been found that ultrasound can cause inactivation of polyphenol oxidase in apple juice [11] and the PME and PG in tomato juice [10,12]. On the other hand, ultrasound treatment can effectively break large particles and improve the properties of juices in terms of viscosity and cloudy stability [13,14]. Moreover, ultrasound can preserve health-promoting functional compounds, and even promote the release of these compounds from the plant matrix, thereby improving the nutritional value of these products [15,16]. Generally, the literature mainly focusses on the ultrasound-assisted processing of freshly squeezed or heat-treated tomato juice. However, industrial tomato paste production is more complex, in which enzyme inactivation and concentration are considered as the most important processing steps to determine its final quality [7]. Unfortunately, there are no literature reports on the ultrasound-assisted processing of industrial tomato paste. Additionally, the underlying mechanisms of the tomato paste viscosity influenced by processing are not well established. In the present work, the effects of ultrasound break at 22 °C (US-Break-22) and 65 °C (US-Break-65) on the viscosity, rheological properties and nutritional values of tomato paste were investigated, compared with those of traditional thermal break at 65 °C (Break-65) and 90 °C (Break-90). The pectin-related enzyme, particle size, and serum pectin were further characterized to clarify the underlying working mechanisms of breaking methods on the viscosity and rheological properties of tomato pastes.

## 2. Materials and Methods

### 2.1. Materials

Tomatoes with uniform size and maturity were purchased from a local market in Chongqing, China. The selected tomatoes were stored at 4 °C before tomato paste production. All-trans lycopene, lutein, β-carotene, galacturonic acid (GalA), arabinose (Ara), lactose (Lac), galactose (Gal), rhamnose (Rha), fucose (Fuc), xylose (Xyl), acetonitrile of chromatography grade, 2,2-diphenyl-1-picrylhydrazyl (DPPH), and 2,2′-azinobis-(3-ethylbenzothiazoline-6-sulfonic acid) (ABTS) were purchased from Sigma Chemical Co. (St. Louis, MO, USA). Rutin, quercetin, naringenin, naringenin-7-*O*-glucoside, protocatechuic acid, ferulic acid, caffeic acid, *p*-coumaric acid, gentistic acid and chlorogenic acid were obtained from Chengdu Biopurify Phytochemicals Co. Ltd. (Chengdu, China). The enzyme-linked immunosorbent assay (ELISA) kit (MEIMIAN) was purchased from Huyu Biological Technology Co., Ltd. (Shanghai, China). The Folin-Ciocalteu’s reagent was obtained from Solarbio Technology Co., Ltd. (Shanghai, China). 1-Phenyl-3-methyl-5-pyrazolone (PMP) was obtained from Adamas Reagent Co., Ltd. (Shanghai, China). Dichloromethane and *n*-butanol of chromatography grade and other chemicals of analytical grade were purchased from Chuandong Chemical Reagent Co., Ltd. (Chongqing, China).

### 2.2. Tomato Paste Preparation

**Figure 1:**
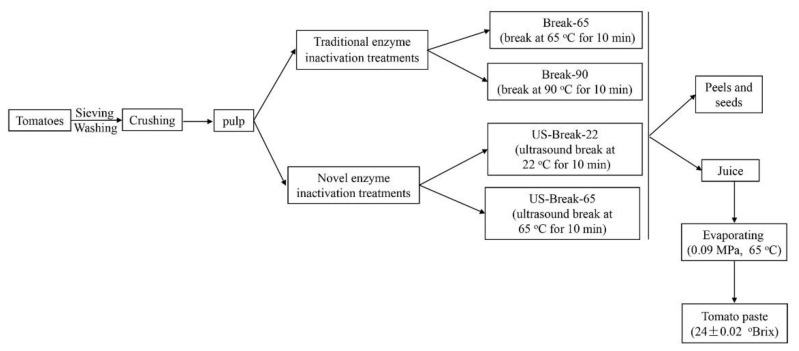
Tomato pastes production scheme. The treatment at 65 °C for 10 min was defined as Break-65. The treatment at 90 °C for 10 min was defined as Break-90. The ultrasound treatment of 87.52 W/cm^2^ at 22 °C for 10 min was defined as US-Break-22. The ultrasound treatments of 87.52 W/cm^2^ at 65 °C for 10 min was defined as US-Break-65.

Generally, the tomato paste preparing process involves several steps, as described in Figure 1. Briefly, the selected tomatoes were washed with tap water and the surface water drops were drained off. Afterward, the tomatoes were cut into pieces and crushed for 2 min in a WBL25B36 domestic juice maker (Midea Group Co., Ltd., Guangzhou, China) to obtain the tomato pulp. The pulp (100 mL) was poured into glass beakers, and then subjected to different break treatments. The thermal treatments at 65 °C and 90 °C water bath for 10 min were named as Break-65 and Break-90, respectively. The glass beaker containing 100 mL of pulp was placed in the inner cavity of the jacket installed on the ultrasonic homogenizer (Ningbo Scientz Biotechnology Co., Ltd., Zhejiang, China). To keep the temperature of the pulp around 22 (room temperature) and 65 °C, the temperature of the circulating water in the jacket controlled by the SDC-6 thermostat (Ningbo Scientz Biotechnology Co., Ltd., Zhejiang, China) was set as 15 and 55 °C, respectively. The titanium probe with a 6 mm diameter was introduced into the homogenate with a depth at 15 mm. The ultrasound homogenizer was operated in a pulse model of 2s-on-2s-off at the power of 500 W. The frequency was approximately 20-25 kHz. The ultrasonic intensity was calculated as 87.52 W/cm^2^ according to the method of Margulis et al. [17]. The treatments at a power of 87.52 W/cm^2^ at 22 and 65 °C for 10 min were named as US-Break-22 and US-Break-65, respectively. The pulp needed pre-heat to the corresponding temperature before all breaking treatments. 

After breaking treatments, the pulp was immediately cooled to room temperature in ice water and filtered through a 60-mesh stainless steel screen (0.25 mm) to remove seeds and peels and obtain tomato juice. The content of total soluble solid of the tomato juice was approximately 4.20 ± 0.02 °Brix. The tomato juice (1000 mL) was then evaporated to achieve total soluble solid content of 24.00 ± 0.02 °Brix under the vacuum of 0.09 MPa at 65 °C for 120 min. 

### 2.3. Physical Characterization of the Tomato Pastes

#### 2.3.1. Particle Size Distribution of the Tomato Pastes

Particle size distribution (PSD) of the tomato pastes was determined by using a Mastersizer 2000 (Malvern Instruments Ltd., Worcestershire, UK) following the method of Tan et al. [18]. The PSD curve was obtained and the area-based (*D*_[3,2]_) and volume-based (*D*_[4,3]_) mean diameters for all samples were also reported.

#### 2.3.2. Rheological Properties of the Tomato Pastes

The rheological properties of the tomato paste were performed using an MCP-302 rheometer (Anton Paar Co., Ltd., Graz, Austria). The instrument was equipped with a cylinder geometry of 29 mm in diameter and 0 mm in gap. The tomato paste (15 g) was carefully loaded into the bottom of the cylinder. The measurements were performed at 25 °C. The loaded samples were equilibrated for 300 s before the measures to relax and attain the desired temperature. The apparent viscosity (mPa‧s) of the tomato paste was determined as the average viscosity at a constant shear rate of 50 s^−1^ for 120 s [19]. In steady shear measurements, the shear rate was increased stepwise from 0.01 s^−^^1^ to 500 s^−1^. The obtained curve of shear stress (*τ*, Pa) against shear rate ($\overset{\cdot }{\gamma }$, s^−1^) was fitted to the Herschel-Bulkley model of *τ = τ*_0_ + *K$\overset{\cdot }{\gamma }$^n^* (herein, *τ*_0_, *K* and *n* refer to the yield stress (Pa), consistency coefficient (Pa·s^n^) and flow behavior index). These parameters were used to quantificationally describe the steady state rheological nature of the tomato paste.

The linear and non-linear rheological properties of the tomato paste was studied in the strain range from 0.01 to 500% at 1 Hz to determine the small amplitude oscillatory shear and large amplitude oscillatory shear (LAOS) behavior [20]. Consequently, the strain amplitude ≤1% was determined as the linear viscoelastic region, while the strain amplitude >1% was determined as the non-linear viscoelastic region (Figure S1). The strain amplitude of 0.1, 1, 10, 100, and 300% was selected to analyze the LOAS behaviors of the tomato paste. Lissajous curves and the stress wave plots were plotted using the software of OriginPro 18.0.

Frequency sweeps were carried out in a range of 0.1–50 Hz at 0.1% strain amplitude. The storage modulus (*G*′), loss modulus (*G*″) were recorded, and the loss tangent (tanδ = *G*″/*G*′) were also obtained. The traces of both moduli were fitted to the power models of *G*′(ω) = *k*′*ω^n^*′ and *G*″(ω) = *k*″*ω^n^*″. Here, *k*′ and *k*″ refer to the consistency coefficients (Pa·s^n^), while *n*′ and *n*″ refer to the behavior indices [21].

### 2.4. Chemical Characterization of the Tomato Pastes

#### 2.4.1. Enzyme Extraction and Analysis

Firstly, the deionized water was added to the tomato pastes and magnetically stirred for 30 min to obtain the mixture with the total solid content of 4.20 ± 0.02 °Brix. Then, 5 g of mixture was added into 10 mL of 50 mmol/L sodium acetate buffer solution (pH 5.5, containing 1.8 mol/L NaCl, 4 °C) and magnetically stirred for 15 min in an ice bath. Then the samples were centrifuged at 12,000× *g* at 4 °C for 30 min (Centrifuge 5810 R, Eppendorf AG, Hamburg, German). The supernatant was collected as the crude enzyme extract and stored at 4 °C until analysis. The residual activity (RA, %) of PME and PG were determined using an enzyme-linked immunosorbent assay as described by the method of Liu et al. [22]. The RA of PME and PG was calculated by the equation of RA (%) = *A_t_*/*A*_0_ × 100 (herein, *A_t_* and *A*_0_ refer to the enzyme activity of tomato pastes and control).

#### 2.4.2. Serum Pectin of the Tomato Pastes

The content of serum pectin was determined following the method of Gao et al. [23] with some modifications. Briefly, 35 mL of pre-heated ethanol (75 °C) was added into 5 g of the serum in a 50 mL-centrifuge tube and heated in a water bath (85 °C) for 10 min with continuous stirring. After cooling, the ethanol was added to make the total volume of 50 mL, and then centrifuged at 4000× *g* for 15 min. The pellet was repeatedly washed with three portions (3 × 20 mL) of 67% ethanol solution in an 85 °C water bath. Subsequently, the pellet obtained was mixed thoroughly with 80 mL of H_2_SO_4_ solution with pH 0.5, and heated in an 85 °C water bath for 60 min. After cooling, the mixture was transferred to a 100 mL volumetric flask and make up to volume with the sulfuric acid solution. The galacturonic acid contents in the above filtrate was calorimetrically determined using a L6 UV-Vis spectrophotometer (INESA (Group) Co., Ltd., Shanghai, China) at 520 nm. The results were expressed as per mg of galacturonic acid in one gram of serum.

The pectin materials in the serum of tomato pastes were extracted according to the method of Chou et al. [24] with some modifications. Briefly, 200 mL deionized water was added to the tomato paste (50 g) and magnetically stirred for 30 min. Then, the mixture was centrifuged at 10,000× *g* at 20 °C for 20 min (Centrifuge 5810 R, Eppendorf AG, Hamburg, German). Then the obtained supernatant was vacuum filtered through 12.5-cm diameter filter paper. The filtrate was mixed with a 10-time volume of 95% ethanol and subsequent stirring of 30 min. The mixture was centrifuged at 3500× *g* for 15 min at 20 °C. The pellet was oven-dried at 40 °C and stored in a desiccator for further analyses. The weight-average molecular weight (*M_w_*), the degree of methoxylation (*DM*) and the monosaccharide composition of the pectin were determined by the method of our previous reports [13].

#### 2.4.3. Ascorbic Acid in the Tomato Pastes

The tomato pastes were freeze-dried into powder for ingredients determination. Ascorbic acid in the freeze-dried samples was quantified by consulting the HPLC method of Adekunte et al. [25]. The results were expressed as mg/100 g dry weight (DW).

#### 2.4.4. Phenolic Compounds in the Tomato Pastes

The phenolic compounds of freeze-dried samples were extracted and determined by the method of Vallverdú-Queralt [26] and modified. Briefly, 2 mL 70% methanol was added into 0.5 g of samples and mixed thoroughly. The mixture was stirred for 30 min at 30 °C in the dark. Then the supernatant was obtained by a centrifugation at 10,000× g for 10 min at 4 °C (Centrifuge 5810 R, Eppendorf AG, Hamburg, German). The pellet was extracted again with the same regime. The combined supernatant was fixed to 5 mL by adding 70% (*v/v*) methanol and filtered through a 0.45 µm membrane filter for further determine the phenolic compounds using HPLC method.

A Shimadzu LC-20A system equipped with a 20-A photodiode-array detector (Shimadzu Co., Kyoto, Japan) was used for phenolic compounds quantification. A C18 reverse-phase column (250 × 4.6 mm i.d., 5 µm) (Thermo Fisher Scientific Co., Ltd., Waltham, MASS, USA) was adopted and kept at 40 °C. The gradient eluent system consisted of 0.1% phosphoric acid solution (*v/v)* (eluent A) and methanol (eluent B). The volume percentage of eluent B was operated as 15–60% (0–30 min) → 60–80% (30–35 min) → 80–90% (35–40 min) → 90–15% (40–50 min) → 15% (50–60 min). The photodiode-array detector was monitored at 280 nm. The injection volume and flow rate were 20 µL and 0.4 mL/min, respectively. The HPLC chromatogram of the standard phenolics mixture are shown in Figure S2. The phenolic content was expressed as dry weight based, per gram of dry weight (DW).

#### 2.4.5. Carotenoids in the Tomato Pastes

The identification and determination of carotenoids in freeze-dried tomato pastes were conducted by the method of our previous report [13]. The results were expressed as mg/100 g dry weight (DW).

### 2.5. Hydrophilic and Lipophilic Antioxidant Activities of the Tomato Pastes

The extract and pellet obtained in 2.4.4 were selected to measure the hydrophilic (HAA) and lipophilic (LAA) antioxidant activities. The extract was diluted 10 times with 70% methanol and then measured the HAA. The pellet was re-extracted with 2 mL of chloroform and stirred at 30 °C for 30 min. Then it was centrifuged at 12,000× *g* for 10 min at 4 °C and the supernatant were separated. The pellet was re-extracted with 2 mL of chloroform in the same conditions. Finally, the supernatants were combined in a volumetric flask and made up to 5 mL with chloroform. Then it was diluted 10 times for LAA determinations. Both HAA and LAA were expressed as the scavenging abilities against the free radicals of DPPH and ABTS [27]. The results were expressed as µmol of TE/100 g dry weight (DW).

### 2.6. Statistical Analyses

All experiments in the present study were conducted in triplicates. The data were analyzed by one-way analysis of variance (ANOVA) followed by Tukey’s comparison test using SPSS 19.0 (IBM, Chicago, IL, USA). The data were presented as mean ± standard deviation (SD). Values were regarded as significantly different when *p* < 0.05.

## 3. Results and Discussion

### 3.1. Effects of Different Breaking Treatments on PME and PG

**Figure 2:**
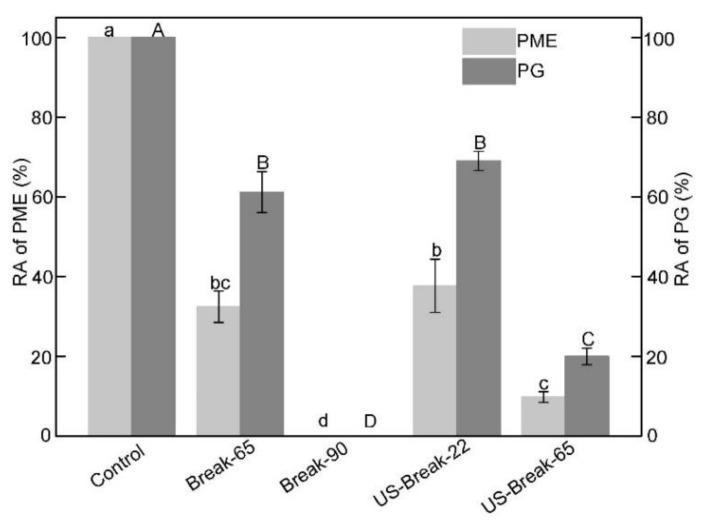
Effects of different breaking treatments on the activity of PME and PG in pastes. RA refers to the residual activities of the enzymes. Control refers to the untreated raw tomato juice. Break-65, Break-90, US-Break-22 and US-Break-65 refer to the thermal break at 65 °C for 10 min, thermal break at 90 °C for 10 min, ultrasound break at 22 °C for 10 min, and ultrasound break at 65 °C for 10 min, respectively. ^a–d^ The RA of the PME data bearing different lowercase letters were significantly different (*p* < 0.05). ^A–D^ The RA of PG data bearing different capital letters were significantly different (*p* < 0.05).

The residual activity (RA) of PME and PG in tomato pastes as affected by different breaking treatments are presented in Figure 2. Obviously, the RA of PME and PG in tomato pastes obtained by Break-65, Break-90, US-Break-22 and US-Break-65 showed significant decrease behaviors compared with control (*p* ˂ 0.05), while the decrease level was dependent on the type of enzymes and temperature. The RA of PME in tomato pastes obtained by Break-65, Break-90, US-Break-22 and US-Break-65 were 32.45, 0.00, 37.68 and 9.78%, respectively. The RA of PG in tomato pastes obtained by Break-65, Break-90, US-Break-22 and US-Break-65 were 61.18, 0.00, 69.03 and 20.00%, respectively. It could be clearly found that the PG was more resistant to thermal and ultrasound than PME, which could be ascribed to the reversible configuration of the PG [28]. Clearly, the RA of both enzymes in the Break-65 paste was significantly higher than that of Break-90 paste. The results showed that the enzymes inactivation increased as the temperature increased [29,30]. The RA of both enzymes in the US-Break-65 paste was significantly lower than that of Break-65 paste and US-Break-22 paste (*p* ˂ 0.05), which indicated that the rate of decrease in residual enzyme activity was higher at a higher ultrasound temperature. Similarly, Wu et al. [9] reported that the residual activities of PME in tomato juice subjected to the thermosonication (24 kHz) at 60 and 65 °C were significantly lower than the values in the thermal-treated samples at the same temperatures and exposure times. It is obvious that cavitation is the essence of ultrasound treatment or processing. It has been reported that one of the major factors in enzyme inactivation is the localized high temperature and pressure caused by the sudden and violent collapse of cavitation bubble [31]. The intensities of cavitation bubble collapse in aqueous solution increased as the ultrasound temperature [9]. So, the thermal and mechanical forces in the US-Break-65 have a synergistic effect on enzyme inactivation [32]. There was no significant difference of the RA of PME and PG in the tomato paste prepared by Break-60 and US-Break-22. Based on the results in Figure S3. This behavior could be attributed to the breakdown of the cell wall by the high shearing effects during cavitation of the ultrasound, resulting in the releasing of enzymes from the cell walls [11]. Due to lack of protection of the cell wall, the released enzyme was easily inactivated during the concentration processing.

### 3.2. Effects of Different Breaking Treatments on the Content and Physico-Chemical Properties of Serum Pectin

#### 3.2.1. Pectin Content

The pectin content was expressed as the amount of GalA in the serum. As shown in Table 1, the level of pectin in the serum of the tomato pastes produced by Break-65, Break-90, US-Break-22 and US-Break-65 were 14.65, 22.85, 26.37 and 31.50 mg GalA/g, respectively. In contrast, the control sample had the lowest pectin content (10.91 mg GalA/g), which indicated that part of the pectin leached out the cell wall during all treatments. The amount of serum pectin in Break-90 paste was significantly higher than that of Break-65 paste (*p* ˂ 0.05), which was in agreement with the previous report [33,34]. Lin et al. [33] reported that the yield of water-soluble pectin in the serum of hot break tomato paste was approximately 1.82 times than that of the cold break paste. This could be attributed to the following reasons. Firstly, the heat treatment with higher temperature could have caused more extensive destruction of the cell wall and released a more wall-bound pectin [35]. Secondly, higher temperature could have led to a larger increase in pectin solubility, attributing to the greater thermo-solubilization and β-eliminative depolymerization of pectin [36]. Thirdly, the serum pectin in Break-90 paste may not have been degraded by the pectin-related enzymes due to the high temperature [34].

The contents of serum pectin in US-Break-22 and US-Break-65 pastes were significantly higher than that of Break-65 and Break-90 pastes. This was attributed to the cavitation effect of ultrasound, which caused a greater deconstruction on the cell wall than the conventional thermal treatments, thereby eliminating the physical constraint of the matrix and finally favoring the extraction of pectin [37]. Besides, it could also be seen that the content of serum pectin in US-Break-65 paste was significantly higher than that of the US-Break-22 paste (*p* ˂ 0.05), attributing to the synergistic effect of ultrasound and heat on the enzyme inactivation and cell wall destruction.

#### 3.2.2. Degree of Methyl-Esterification (*DM*) of Serum Pectin

The average *DM* values of the pectin are shown in Table 1. The serum pectin of tomato pastes was characterized as low methyl-esterification pectin with a degree of *DM* of 19.53–36.63%. Generally, PME catalyzed the removal of the methyl groups in the linear galacturonic acid-rich domain. Then the de-esterified pectin molecule was de-polymerized by PG, which finally caused de-polymerization of serum pectin in tomato product [8]. The endogenous PME and PG were completely inactivated by Break-90, resulting in that the pectin had a comparable *DM* value with the control [38]. However, the *DM* of pectin in Break-65 paste was lower than that in Break-90 paste, attributing to the action of PME in Break-65 paste. The similar results obtained by Santanina et al. [36] showed that the *DM* of the serum pectin was apparently decreased after the carrot purées treated by 60 °C for 45 min, compared with the samples treated at 95 °C for 45 min. It was also observed that the serum pectin in US-Break-65 paste had a higher *DM* than that in Break-65 paste and US-Break-22 paste, probably due to more PME inactivation induced by US-Break-65 than Break-65 and US-Break-22. Interestingly, there were no significant differences in the *DM* values of serum pectin in Break-65 paste and Break-22 pastes (*p* > 0.05). It was related to the fact that the released enzyme caused by ultrasound cavitation lacked the protection of the cell wall, causing its inactivation during concentration processing.

#### 3.2.3. Average Molecular Weight (Mw) of Serum Pectin

As showed in Table 1, the *M_w_* of Break-65 paste (67.27 kDa) and Break-90 paste (161.23 kDa) were significantly lower than that of control (196.10 kDa) (*p* < 0.05), indicating that heat treatment caused the degradation of pectin [39]. By contrast, the *M_w_* of serum pectin in pastes produced by Break-65, US-Break-22 and US-Break-65 were significantly lower than the value of pastes produced by Break-90 (*p* < 0.05), ascribing to the fact that the endogenous PG depolymerized the de-methylesterified pectin, finally resulting in a drastically decrease of *M_w_* [8,33]. Although CB caused more inactivation of PME and PG than UT, there was no significant difference in the *M_w_* values of CBPP and UTPP (*p* > 0.05).

#### 3.2.4. Chemical Properties of Serum Pectin

The monosaccharide composition of serum pectin from differently prepared tomato paste is shown in Table 1. It can be observed that GalA is the major sugar in the serum pectin of all samples, followed by Gal, Xyl, Rha, Ara, Man and Fuc, respectively. Man may originate from the non-pectic polysaccharides such as residual starch, glucomannan and non-crystalline cellulose [40]. Notably, the types of monosaccharide in pectin did not change by different breaking treatments, but their contents changed. The contents of GalA in the serum pectin of tomato pastes produced by Break-65, Break-90, US-Break-22 and US-Break-65 were 49.22, 69.74, 49.59 and 59.66 mol%, respectively. The content of neutral sugar in the serum pectin of tomato pastes produced by Break-65, Break-90, US-Break-22 and US-Break-65 were 50.14, 29.79, 49.94 and 39.87 mol%, respectively. These results indicated that the serum pectin of pastes produced by Break-90 and US-Break-65 were rich in GalA, while the serum pectin of the pastes produced by Break-65 and US-Break-22 were rich in neutral sugars.

In view of the structure of pectin molecules, GalA makes the most considerable contribution to the backbone of homogalacturonan (HG) and rhamnogalacturonan (RG) regions, and the Rha constitutes the rhamnogalacturonan-I (RG-I) backbone region with GalA. The Gal and Ara represented the main neutral sugars of the side chains of RG-I, while Fuc and Xyl mainly presented in the side chain of rhamnogalacturonan-Ⅱ (RG-Ⅱ) and xylogalacturonan (XG) or apiogalacturonan (APG), respectively. The molar ratios of the monosaccharides were also determined to gain insight into the linearity/branching of pectin. The molar ratio of GalA/(Rha + Ara + Gal + Xyl) reflects the linearity of pectin, in which a high value suggested the presence of linear pectin/HG-rich pectin. The molar ratio of (Ara + Gal)/Rha represents the average size of the neutral side chains [38].

The molar ratio of GalA and Rha in serum pectin of Break-90 paste was lower than that of control, indicating that heat induced the cleavage of HG and RG-I backbone. In contrast, the molar ratio of Gal and Ara was significantly higher than that of the control (*p* ˂ 0.05). It could be concluded that the cleavage of RG-I backbone induced by heat mainly occurred in the zones with less side chains or shorter chains. The linearity of the serum pectin of Break-90 paste was significantly lower than that of the control (*p* ˂ 0.05), while the average size of the neutral side chains had the opposite trend. It was further demonstrated that heat mainly caused the degradation of the backbone of serum pectin [41]. Compared with the pectin of Break-90 paste, the pectin of pastes produced by Break-65, US-Break-22 and US-Break-65 had lower molar ratios of GalA and higher molar ratios of Rha, Gal, Xyl and Ara. The pectin of Break-90 paste had a significantly larger linearity and shorter neutral side chain than that of Break-65 paste, US-Break-22 paste, and US-Break-65 paste (*p* ˂ 0.05). This result indicated that the pectin-degradation induced by the enzymes occurred mainly in the HG region of pectin [42].

In summary, the contents of serum pectin of tomato pastes followed the order of US-Break-65 > US-Break-22 > Break-90 > Break-65, while the order of *DM* and *M_w_* were Break-90 > US-Break-65 > Break-65 ≈ US-Break-22. The molar ratio of GalA that represented the linearity of the pectin followed the order of Break-65 > US-Break-65 > Break-65 ≈ US-Break-22. The pectin rich in the linearity backbone has a greater viscosity and stronger intermolecular interaction than that rich in branched side chains [43]. However, a decrease of GalA ratio could promote the flexibility of pectin molecules, and it also could increase the kinking effects of Rha residues in the molecular conformation [33]. It could be inferred that the order of pectin viscosity and the three-dimensional network structure strength had the same trend, namely Break-90 > US-Break-65 > Break-65 ≈ US-Break-22. These results might be closely related to the properties of tomato pastes, including viscosity and rheological properties.

### 3.3. Effects of Different Breaking Treatments on Particle Size Distribution of Tomato Pastes

**Figure 3:**
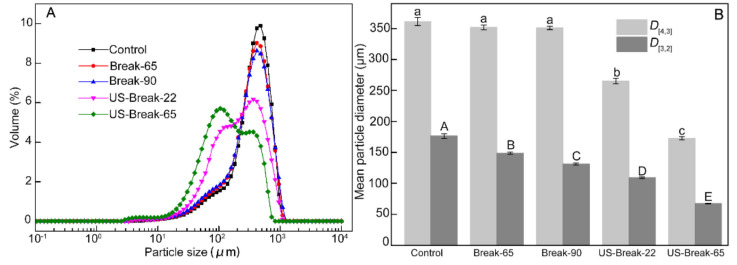
Effects of different breaking treatments on particle size distribution (**A**) and mean particle diameter size (**B**) of tomato paste. Break-65, Break-90, US-Break-22 and US-Break-65 refer to the thermal break at 65 °C for 10 min, thermal break at 90 °C for 10 min, ultrasound break at 22 °C for 10 min, and ultrasound break at 65 °C for 10 min, respectively. *D*_[4,3]_ and *D*_[3,2]_ represent the volume-based mean diameter and area-based mean diameter, respectively. ^a–c^ *D*_[4,3]_ data bearing different lowercase letters were significantly different (*p* < 0.05). ^A–E^ *D*_[3,2]_ data bearing capital letters were significantly different (*p* < 0.05).

The effects of different treatments on the particle size distribution of tomato pastes are shown in Figure 3. Clearly, the unimodal PSD were obtained for control, Break-65 paste, and Break-90 paste. In contrast, both US-Break-22 paste, and US-Break-65 paste were displayed as bimodal curves with two peaks around 400 μm and 100 μm (Figure 3A). Obviously, with the ultrasound temperature increased from (US-Break-22) to 65 °C (US-Break-65), the peak intensity at 400 μm decreased, while the peak intensity at 100 μm increased significantly (*p* ˂ 0.05). More specifically, the data in Figure 3B claimed that *D*_[3,2]_ of the pastes produced by Break-65 and Break-90 were significantly lower than the control (*p* ˂ 0.05), while there were no significant differences of *D*_[4,3]_ between them (*p* > 0.05). It was also observed that the *D*_[3,2]_ of Break-90 paste was smaller than that of Break-65 paste, which indicated that Break-90 paste has a greater proportion of small particles. The decrease of particle size of tomato pastes produced by Break-65 and Break-90 could be explained by the fact that thermal treatment could cause the breakdown of the cell wall, and a higher temperature, a greater damage [10]. In considering the implications of *D*_[4,3]_ and *D*_[3,2]_, these results attested that the thermal treatment brought about more serious structural changes for smaller particles than the larger ones [44].

The *D*_[4,3]_ and *D*_[3,2]_ of the US-Break-22 and US-Break-65 pastes were significantly lower than those of Break-65 and Break-90 pastes (*p* ˂ 0.05). More importantly, both *D*_[3,2]_ and *D*_[4,3]_ of US-Break-65 paste were significantly lower than those of US-Break-22 paste, and the *D*_[4,3]_ showed a more rapid decrease compared with the *D*_[3,2]_ (*p* ˂ 0.05). It implied that the ultrasound-induced more serious structural changes for larger particles than the smaller ones [13]. It could also be seen that ultrasound and heat had a synergistic destruction effect on the particles. In this sense, the present US-Break-22 and US-Break-65 induced an obvious homogenization effect on the tomato paste. This homogenization effect caused by ultrasound could be explained by the cavitation and the shear stress generated by ultrasound wave propagation [9].

### 3.4. Effects of Different Breaking Treatments on the Viscosity and Rheological Properties of Tomato Pastes

#### 3.4.1. The Viscosity of Tomato Pastes

**Figure 4:**
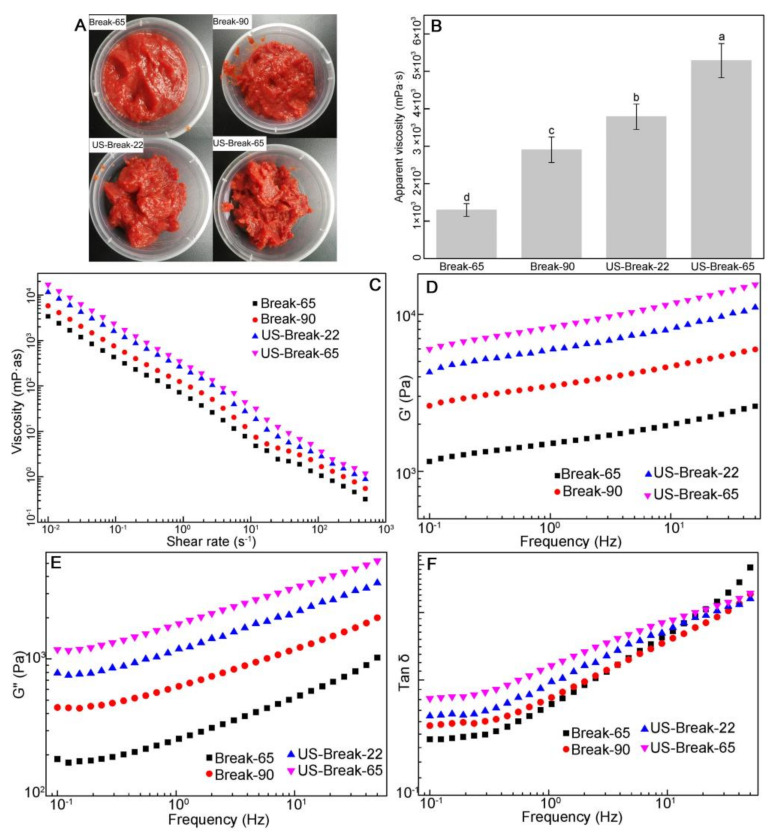
Effects of different breaking treatments on the appearance (**A**), viscosity (**B**), flow behavior (**C**), the dynamic rheological properties (**D**–**F**) of tomato paste. Break-65, Break-90, US-Break-22 and US-Break-65 refer to the thermal break at 65 °C for 10 min, thermal break at 90 °C for 10 min, ultrasound break at 22 °C for 10 min, and ultrasound break at 65 °C for 10 min, respectively ^a–d^ Data bearing different lowercase letters were significantly different (*p* < 0.05).

The appearance and viscosity of different tomato pastes are shown in Figure 4A,B, respectively. The viscosity followed an order of US-Break-65 paste > US-Break-22 > Break-90 > Break-65. For the conventional heat-treated samples, the viscosity of Break-90 paste was significantly larger than the Break-65 paste (*p* ˂ 0.05). The similar results reported by Hsu [28] indicated that the tomato juice prepared by hot break (92 °C-2 min) had a significantly higher viscosity than the tomato juice prepared by cold break (60 °C-2 min). For the ultrasound-treated samples, the viscosity of US-Break-22 paste, and US-Break-65 paste were significantly higher than that of Break-65 paste and Break-90 paste (*p* ˂ 0.05). The increase in viscosity due to ultrasound has been verified in other juices or puree such as tomato juice [45] and avocado puree [46]. Wu et al. [9] found the viscosity of the tomato juice treated by thermosonication at 60–70 °C was 2–4-fold larger than the heat treated or untreated samples. The viscosity of US-Break-65 paste was greater than that of US-Break-22 paste, indicating that heat-ultrasound has a synergistic effect on the viscosity of tomato paste.

#### 3.4.2. The Rheological Properties of the Tomato Pastes

##### Flow Behavior of the Tomato Pastes

The effects of different breaking treatments on the flow behavior of tomato paste are shown in Figure 4C. Clearly, the apparent viscosity of differently tomato pastes significantly decreased as the shear rate increased from 0.01 to 500 s^−1^. It suggested that the tomato paste was a non-Newtonian fluid with a shear-thinning or pseudoplastic behavior. The data in Figure 4C were fitted to the Herschel-Bulkley model, and the flow behaviors of the pastes were quantitatively described in Table S1. All samples presented a flow behavior (*n*) value ≤0.50, which further confirmed their shear-thinning nature. Regarding the yield stress (*τ*_0_) and consistency coefficient (*K*), both of them followed the same order of US-Break-65 paste > US-Break-22 paste > Break-90 paste > Break-65 paste. However, the *n* followed the opposite order of US-Break-65 paste ˂ US-Break-22 paste ˂ Break-90 paste ˂ Break-65 paste. These facts declared that the integrity of the network or the solid-like nature of the pastes followed the order of US-Break-65 paste > US-Break-22 paste > Break-90 paste > Break-65 paste. Fito et al. [47] reported that hot break tomato concentrate had a higher apparent viscosity and *K* value and lower *n* value than cold break samples. It was attributed to the fact that the enzyme inactivation during hot break processing could further prevent pectin degradation. Vercet et al. [32] reported that the apparent viscosity and yield stress of tomato juice treated by ultrasound with 20 kHz-117 µm amplitude at 70 °C were 1.6 and 2.2-fold higher than the values for thermal treatment, respectively. They also declared that the ultrasound treated samples had a stronger interaction between the components than that in thermal-treated samples.

##### Dynamic Rheological Properties of the Tomato Pastes

As shown in Figure 4D–F, the viscoelastic behavior, including storage modulus (*G*′) and loss modulus (*G*″) of the tomato pastes were determined at the 0.1~50 Hz in the frequency sweep model. *G*′ was always higher than *G*″ for all samples, and both moduli were continuously increased with frequency. It was consistent with the fact that the network structure of the tomato paste consisted of solid particles suspended in pectin serum [20]. It was also revealed that the tomato paste has a weak gel-like behavior. Furthermore, by judging the fact that *n*″ > *n*′ (Table S1), *G*″ exhibited a higher frequency dependence than *G*′, indicating that the viscidity increased faster than the elasticity in all samples. The loss tangent (tan *δ*) of all pastes increased with frequency, which matched with their shear-thinning behaviors observed in steady shear measurements. The highest *G*′ and *G*″ were observed in US-Break-65 paste, followed by US-Break-22 paste, Break-90 paste, and Break-65 paste. In this sense, US-Break-65 paste displayed the maximum shear-thinning upon gentle oscillations. The Break-90 paste had larger *G*′ and *G*″ than Break-65 paste, indicating that the former had a better viscidity and elasticity than the later. The similar results obtained by Sánchez et al. [48] found that the *G*′ and *G*″ of the tomato paste treated by 80 °C were larger than those treated by 65 °C. It could be attributed to the fact that tomato paste showed a lower enzyme activation, higher water-insoluble solids content and smaller particle size with the increased of break temperature. The *G*′ and *G*″ of US-Break-65 paste were greater than that of US-Break-22 paste, indicating that thermal and ultrasound had a synergistic effect on the viscoelastic behavior of tomato paste.

##### Large-Amplitude Oscillation Shear (LAOS) Behavior of the Tomato Pastes

Generally, the linear rheological properties of foods are usually measured by a small-amplitude oscillation shear test. However, this method failed to describe the nonlinear rheological properties that occurred during in food processing and chewing. Recently, the large amplitude oscillation shear (LAOS) test has been widely used to describe the nonlinear rheological behaviors of complex fluid systems [49].

As illustrated in Figure S1, *G*′ and *G*″ showed a decrease trend with the increase in strain amplitude in the nonlinear region, which indicated that all the tomato pastes exhibited a typical LAOS type Ⅰ-strain thinning behavior [50]. It implied that the network junctions of the polymer chain were broken at large strain amplitude, and the chains failed to retain the network structure. Then the broken fragments might form new network structure with less resistance against the deformation caused by large strain amplitude, leading to the strain thinning behavior of materials. The similar results were reported by Duvarci et al. [20], who demonstrated that the tomato paste had a strain thinning behavior regardless of frequency. In the present study, in the linear regime of strain ≤1%, both moduli remained constant and the value of *G*′ were larger than the *G*″, indicating that the tomato paste showed an elasticity-dominated behavior. However, *G*′ and *G*″ at strain of about 10% had a crossover, and then the value of *G*″ was larger than the *G*′, which indicated that the tomato paste showed a viscosity-dominated behavior. In both linear and nonlinear regime, US-Break-65 paste had the largest *G*′ and *G*″, followed by US-Break-22 paste, Break-90 paste, and Break-65 paste. It was possibly inferred that the order of elasticity and viscosity of tomato pastes showed the same trend in the order of US-Break-65 paste > US-Break-22 paste > Break-90 paste > Break-65 paste. Further information was discovered by analyzed the Fourier-transform (FT) rheology and Lissajous curves.

**Figure 5:**
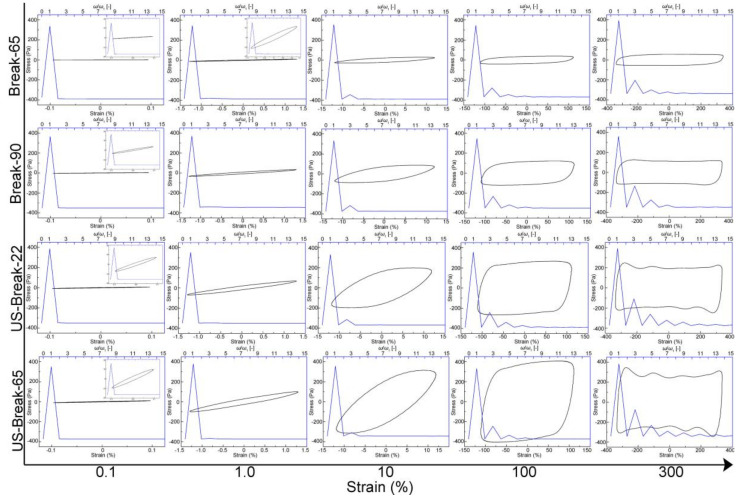
Effects of different breaking treatments on the Lissajous curves (black closed curve). Break-90, US-Break-22 and US-Break-65 refer to the thermal break at 65 °C for 10 min, thermal break at 90 °C for 10 min, ultrasound break at 22 °C for 10 min, and ultrasound break at 65 °C for 10 min, respectively.

Figure 5 showed the FT rheology spectra of four different tomato pastes (blue curve). It was clearly found that the stress response was reflected as the first harmonic at the strain less than 1%, which indicated that the tomato paste has a linear viscoelastic behavior. However, the odd harmonics (3, 5, 7…) appeared when the strain greater than 1%, indicating that the tomato paste showed a nonlinear viscoelastic behavior [49]. The relative intensity of the third harmonic to the first harmonic (*I_3/1_*) has been used to quantify nonlinearity in the stress response [51]. As shown in Figure S4, the *I_3/1_* of four tomato pastes remained constantly with strain ≤1% and increased dramatically with a higher strain. These results showed that the nonlinear viscoelastic behavior of tomato paste increased as the strain increased. It was also observed that the order of nonlinear viscoelastic behavior was US-Break-65 paste > US-Break-22 paste > Break-90 paste > Break-65 paste.

The system behavior and structural transitions of complex fluid under larger deformation could be analyzed by the plots of stress versus strain (Lissajous plots). Figure 5 showed the Lissajous figures for all tomato paste samples at different strains (black closed curve). The changes of rheological behaviors from linear to nonlinear regime can be visually illustrated by the different shapes of the Lissajous curves. In the linear regime, the Lissajous plots were perfectly elliptical, indicating that the tomato paste showed an ideal viscoelastic behavior. The area of Lissajous plots decreased in the order of US-Break-65 paste > US-Break-22 paste > Break-90 paste > Break-65 paste in the linear regime, indicating that the US-Break-65 paste showed the strongest elastic nature. In the nonlinear regime, the shapes of Lissajous plots changed from an ellipse to a parallelogram, indicating an increased viscous dissipation and a shift from elastic-dominated to viscous-dominated behavior [52]. For all strains, the areas of the Lissajous plots decreased in the order of US-Break-65 paste > US-Break-22 paste > Break-90 paste > Break-65 paste. This was related to the serum pectin in the tomato paste, which partially held its microstructure at the non-linear region [24]. The pectin in the tomato paste serum has adhesiveness and flexibility, promoting the interconnection of solid particles. Meanwhile, the lubrication offered by serum pectin could facilitate the progressive orientation within the structure [20].

#### 3.4.3. The Mechanism Underling the Effects of Different Breaking Treatments on the Viscosity and Rheological Properties of Tomato Pastes

Generally, tomato paste can be considered as a two-phase suspension consisting of insoluble particles in a continuous serum with pectin, sugars and organic acids dissolved in it. The viscosity and rheological behavior of differently processed tomato paste might be affected by several factors, such as the concentration, size and morphology of insoluble particles, and the content and physical-chemical properties of the pectin material in its serum. Raviyan et al. [29] and Giner et al. [53] reported that the pectin in serum was degraded by endogenous PME and PG, it could cause the viscosity of tomato product was decreased. Wu et al. [44] declared that the viscosity of hot- and cold-break tomato paste was determined by the particle size. Lin et al. [33] found that the content of pectin in the serum and its physical-chemical properties were the determined factors for the viscosity of hot- and cold- break tomato paste. It could be seen that the underlying mechanism of processing effects for the viscosity of tomato paste deserve further discussion.

In the present study, Pearson’s correlation analyses were used to reveal the mechanism underlying the fact that the effects of different breaking treatments on the viscosity and rheological properties (*K*, *τ*_0_, *G*′, *G*″, solid-like nature, LAOS behavior) of the tomato pastes. The contribution of the particle size and serum pectin-related properties including the content, *DM*, *M_w_*, linearity and side chain length to the viscosity and rheological properties of the tomato paste were analyzed. The results are summarized in Table S2. The results suggested that the particle size (absolute correlation coefficient ≥ 0.920) and the content of pectin in the serum (absolute correlation coefficient ≥ 0.948) are two decisive factors for the viscosity and rheological properties of tomato paste. It could also be found that the influences of *DM*, *M_w_*, linearity and side-chain length of the pectin on the viscosity and rheological properties of ketchup could be ignored. For the insoluble particle phase, the decreased particle size of tomato paste could yield a larger interfacial area, resulting in a strong interaction between particles [9,54]. Moreover, smaller particles could be better dispersed in the pectin network, thereby enhancing the network structure and improving the viscosity of the system [9,32]. For the serum phase, with the pectin increase, the viscosity of the serum increased, and the particles could be better embedded in the network structure formed by pectin at the same time. Therefore, the tomato pastes with smaller particle size and higher serum pectin content had greater viscosity and rheological properties.

### 3.5. Effects of Different Breaking Treatments on the Nutritional Qualities of Tomato Pastes

#### 3.5.1. Levels of Ascorbic Acid

**Figure 6:**
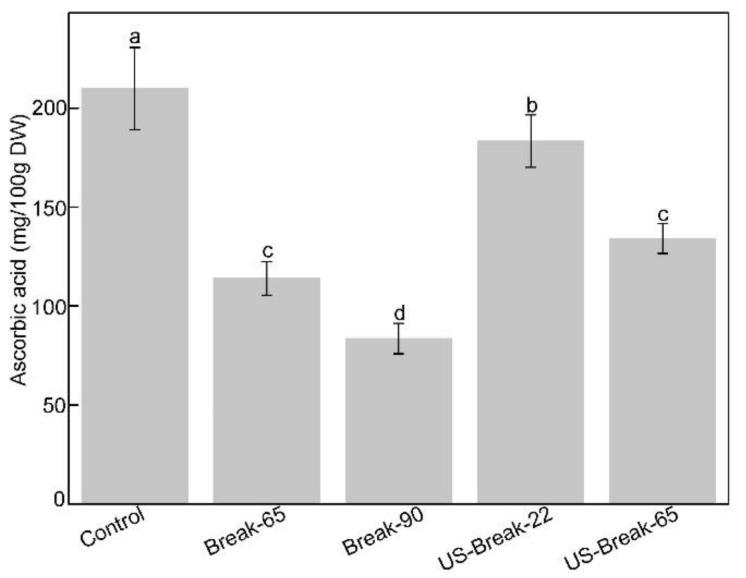
Effects of different breaking treatments on the content of ascorbic acid in tomato juices and pastes. Control refers to the untreated raw tomato juice. Break-65, Break-90, US-Break-22 and US-Break-65 refer to the thermal break at 65 °C for 10 min, thermal break at 90 °C for 10 min, ultrasound break at 22 °C for 10 min, and ultrasound break at 65 °C for 10 min, respectively. ^a–d^ Data bearing different lowercase letters were significantly different (*p* < 0.05).

Ascorbic acid is a key hydrophilic antioxidant micronutrient in tomatoes. It was of great significance to study the influence of different processing processes on ascorbic acid due to its high sensibility to the heat and oxygen. As shown in Figure 6, the levels of ascorbic acid in control, Break-65 paste, Break-90 paste, US-Break-22, US-Break-65 paste were determined as 209.80, 113,93, 83.52, 183.25, and 133.98 mg/100 g DW, respectively. Compared with the control, the level of ascorbic acid in Break-65 paste and Break-90 paste was reduced by 45.70 and 60.19%, respectively. This could be attributed to the fact that although thermal treatment could cause the damage to the cell wall and enhance the release of materials inside the cell wall, the ascorbic acid is also more prone to oxidative degradation under heat treatment, especially at a high temperature [3]. The content of ascorbic acid in US-Break-22 paste was significantly higher than the value of control (*p* < 0.05). This behavior could be explained by the fact that the cell wall was destructed by ultrasound cavitation, and bioactive compounds were released from plant matrixes [55]. Abid et al. [56] reported that ultrasound treatment at 20 °C resulted in a significant increase in ascorbic acid of apple juice. However, heat and hydroxyl radicals produced by the cavitation of ultrasound could cause degradation of ascorbic acid at higher temperatures [57]. Therefore, it was found that the level of ascorbic acid in US-Break-22 paste was significantly higher than the value of US-Break-65 paste (*p* < 0.05). The level of ascorbic acid in US-Break-65 paste was higher than the value of Break-65 pastes, attributing to the fact that the release of ascorbic acid by ultrasound was greater than the degradation caused by the heat and free radicals. Do Amaral Souza et al. [58] reported the similar results that the content of ascorbic acid in camu-camu nectars treated by ultrasound at 40 °C was higher than the untreated samples. They also found that the ascorbic acid was gradually decreased as the ultrasound temperature increased from 40 to 60 °C, while the level of ascorbic acid of samples treated by ultrasound was always larger than that of conventional pasteurization.

#### 3.5.2. Levels of Phenolics Compounds

The changes in phenolic compounds of the tomato pastes are shown in Table 2. Generally, a total of 10 compounds including four flavonols and flavanone (rutin, quercetin, naringenin, naringenin-7-*O*-glucoside) and six phenolic acids (protocatechuic acid, ferulic acid, caffeic acid, *p*-coumsric acid, gentistic acid, chlorogenic acid) were quantified in the present study. Obviously, rutin was identified as the most abundant phenolic compound in all samples, which was consistent with the results obtained by Kelebek et al. [10]. The amounts of total phenolic compounds in control, Break-65 paste, Break-90 paste, US-Break-22 paste, and US-Break-65 paste were detected as 382.20, 419.76, 444.30, 494.23, and 456.55 µg/g DW, respectively.

Compared with the control, all tomato pastes showed higher levels of phenolic monomers and total phenolic compounds. The phenolic compounds increased after the conventional thermal treatments (Break-65 and Break-90), attributing to the cell wall disruption by thermal and promoting the release of these compounds. Moreover, the release increased as the temperature increased [10,59]. The level of total phenolic acid in Break-65 paste and Break-90 paste increased by 15.55% and 2.00%, respectively, and the level of total flavonols/flavanone increased by 8.4% and 19.81%, respectively. The similar results obtained by Kelebek et al. [10] that the tomato paste prepared by cold break method (60–70 °C) showed a higher increase in phenolic acids than those prepared by hot break method (85–90 °C). They also found that cold break paste was characterized by the phenolic acids, while hot break paste was characterized by flavanols and flavanones. The levels of total phenolic acid in US-Break-22 paste and US-Break-65 paste were 100.69 and 94.88 µg/g DW, while the level of total flavonols and flavanone were 393.54 and 361.67 µg/g DW, respectively, which were significantly higher than those in Break-65 paste, and Break-90 paste. It was attributed to the pressures of liquid change rapidly by the shear forces produced during the cavitation of sonication, which could cause disruption of the cell wall and finally promote the bioactive compounds released [55,60]. Abid et al. [61] reported that the total phenolic content of apple juice was increased significantly at a lower ultrasound processing temperature of 20 °C compared with untreated samples. The level of total phenolic acid, total flavonols/flavanone in US-Break-22 paste was higher than those of US-Break-65 paste, attributing to the degradation of phenolic compounds, which increased as the ultrasound temperature increased. Abid et al. [61] reported that the total phenolic content of the apple juice showed a decreased trend with the ultrasound temperature increased from 20 to 60 °C. Rawson et al. [62] also reported that the total phenolic content of watermelon juice decreased as the ultrasound temperature increased from 25 to 45 °C. In general, compared with the control, the level of total phenolic in Break-65 paste, Break-90 paste, US-Break-22 paste, and US-Break-65 paste increased by 9.83, 16.25, 29.31, and 19.45%, respectively. In other words, the mechanical and thermal effects during tomato paste processing could promote the release of phenolic compounds from the cell matrix [63,64].

#### 3.5.3. Levels of Carotenoids

The determined levels of total carotenoids, total lutein, total *β*-carotene and total lycopene, as well as their monomers in tomato pastes, are shown in Table 3. Clearly, the contents of total lycopene, total carotene and total lutein in control were 123.74, 21.90 and 4.5 mg/100g DW, respectively. These results indicated that the lycopene including all-trans- and cis- was the most abundant carotenoid in tomato [65]. Compared with the control, the total carotenoid in the Break-65 and Break-90 paste was decreased by 8.03 and 11.89%, respectively. This was attributed to the oxidation or isomerization of carotenoids caused by thermal treatments [66,67]. Koh et al. [68] reported that the loss of lycopene mainly occurred in the evaporation and sterilization stage during tomato paste processing. The total carotenoid in US-Break-22 and US-Break-65 pastes were significantly higher than that of the Break-65 and Break-90 pastes. It could be further confirmed that the ultrasound can cause damage to the cell wall structure and further promote the release of carotenoids [55,60,66,69]. The total carotenoid in US-Break-22 paste and US-Break-65 paste were comparable with the control. It was attributed to the fact that, although the ultrasound cavitation could promote the release of carotenoids from the cell matrix, the evaporation processing could also cause carotenoids to be degraded or isomerized. Interestingly, the levels of total cis-carotenoids in control, Break-65 paste, Break-90 paste, US-Break-22 paste, and US-Break-65 paste were 18.10, 21.15, 23.64, 25.31 and 25.73 mg/100 g DW, respectively. Combined with the results of Table S3, it was clearly found that the evaporation processing could be resulted in trans-cis geometrical isomerization of the carotenoids [70]. Shi et al. [66] observed that the cis-isomer lycopene in tomato purée treated at 60 °C for 3 h increased by 18.8% compared with the untreated sample. Dewanto et al. [69] also reported that the level of total cis lycopene of tomato slurry increased by 34.88% after treated at 88 °C for 30 min. It is generally known that the cis lycopene shows more bioavailability than its all-trans-isomers [71]. Therefore, it could be concluded that the present US-Break-22 and US-Break-65 can not only retain the carotenoids effectively, but may also improve the biological activity of tomato paste.

#### 3.5.4. Antioxidant Activity of the Tomato Pastes

The effects of different breaking treatments on the lipophilic (LAA) and hydrophilic (HAA) antioxidant activity of pastes were determined in terms of their scavenging capacities to DPPH and ABTS radicals. Generally, ascorbic acid and phenolics are the main water-soluble antioxidants in tomato, while carotenoids are the predominant liposoluble antioxidants [27,72]. Therefore, it could be considered that the intensities of HAA and LAA were decided by the extractable hydrophilic and lipophilic antioxidants in juices or pastes, respectively. As shown in Table 4, there was no significant difference of the HAA between Break-65 paste and Break-90 paste (*p* > 0.05). This could be attributed to the fact that thermal treatments can cause the degradation of ascorbic acid but promote the release of phenolic compounds simultaneously. The HAA of both US-Break-22 paste and US-Break-65 paste were higher than that of Break-65 paste and Break-90 paste, which contributed by the release of ascorbic acid and phenolics caused by ultrasound cavitation. The similar results reported by Abid et al. [56] showed that the apple juice treated by ultrasound showed a stronger antioxidant capacity, attributing to the extracted ascorbic acid and polyphenolic compounds during ultrasound processing. Although the carotenoids were degraded during processing, the LAA of all tomato pastes was still greater than that of the control. It was attributed to the fact that all-trans carotenoids were converted into cis isomers with higher biological activity. The similar results reported by Tomas et al. [73] indicated that the industrial processing paste had a higher total antioxidant capacity than initial tomato fruit. In summary, the US-Break-22 paste showed the best antioxidant capacity, followed by the US-Break-65 paste, Break-90 paste, and Break-65 paste. These results suggested that the breaking treatments by ultrasound not only improved the viscosity and rheological properties of tomato pastes, but also promoted its antioxidant activity.

## 4. Conclusions

Based on the results of apparent viscosity, rheological properties and nutritional values of the tomato pastes, US-Break-22 and US-Break-65 can be considered as potential alternative technologies for conventional Break-65 and Break-90. US-Break-65 paste showed the highest apparent viscosity and rheological properties, followed by US-Break-22 paste, Break-90 paste, and Break-65 paste. The rheological properties included *K*, *τ*_0_, *G*′, *G*″, solid-like nature and LAOS behavior. Interestingly, the present study revealed that the reduction in particle size and the release of pectin into serum induced by breaking treatments were considered as the two fateful factors for the viscosity and rheological properties of the tomato pastes. The levels of ascorbic acid, phenolics, carotenoids, and antioxidant activities of tomato pastes prepared by US-Break-22 and US-Break-65 were significantly higher than those prepared by Break-22 and Break-65. It is concluded that the tomato pastes obtained by ultrasound assisted processing has the advantages of high viscosity, rheological properties and nutritional value. However, these properties of tomato paste depend on the ultrasound parameter. In general, the lower the ultrasound temperature, the lower the viscosity and the better nutritional value. The findings in the present study provide a better understanding of ultrasound for improving viscosity, rheological properties and nutritional characteristics of tomato paste. Further studies should be conducted to elucidate the flavor and the storage stability of US-Break-22 paste and US-Break-65 paste, as well as to develop industrial applications for tomato paste by ultrasound.

## Figures and Tables

**Table 1:** Effects of different breaking treatments on the content and physico-chemical properties of the pectin fractions from the serum of tomato paste.

Properties	Control	Break-65	Break-90	US-Break-22	US-Break-65
pectin content (mg GalA/g serum)	10.91 ± 0.85 ^e^	14.65 ± 1.11 ^d^	22.85 ± 2.17 ^c^	26.37 ± 1.33 ^b^	31.50 ± 3.81 ^a^
GalA(mol%)	81.42 ± 0.85 ^a^	49.22 ± 0.59 ^d^	69.74 ± 2.20 ^b^	49.59 ± 1.01 ^d^	59.66 ± 0.56 ^c^
Fuc(mol%)	0.37 ± 0.02 ^c^	0.65 ± 0.03 ^a^	0.40 ± 0.04 ^c^	0.66 ± 0.05 ^a^	0.52 ± 0.03 ^b^
Rha(mol%)	3.00 ± 0.29 ^c^	5.47 ± 0.31 ^a^	2.46 ± 0.22 ^c^	5.72 ± 0.24 ^a^	4.09 ± 0.22 ^b^
Gal(mol%)	8.36 ± 0.76 ^d^	26.83 ± 1.02 ^a^	15.44 ± 0.20 ^b^	26.73 ± 1.44 ^c^	21.15 ± 0.20 ^b^
Xyl(mol%)	3.25 ± 0.20 ^c^	8.44 ± 0.31 ^a^	6.11 ± 0.39 ^b^	8.17 ± 0.40 ^a^	6.67 ± 0.30 ^b^
Ara(mol%)	2.96 ± 0.22 ^d^	8.93 ± 0.24 ^a^	5.27 ± 0.28 ^c^	8.67 ± 0.56 ^a^	7.45 ± 0.30 ^b^
Man(mol%)	0.64 ± 0.03 ^a^	0.47 ± 0.01 ^c^	0.57 ± 0.01 ^b^	0.47 ± 0.02 ^c^	0.47 ± 0.01 ^c^
Linearity	4.55 ± 0.26 ^a^	0.98 ± 0.02 ^d^	2.36 ± 0.26 ^b^	0.99 ± 0.44 ^d^	1.50 ± 0.04 ^c^
Side chain length	3.78 ± 0.31 ^d^	6.56 ± 0.50 ^bc^	8.42 ± 0.10 ^a^	6.18 ± 0.23 ^c^	7.00 ± 0.12 ^b^
*DM*	34.39 ± 1.41 ^a^	20.89 ± 1.71 ^c^	36.63 ± 2.30 ^a^	19.53 ± 1.43 ^c^	27.75 ± 2.91 ^b^
*M_w_*	196.10 ± 7.57 ^a^	67.27 ± 9.77 ^d^	161.23 ± 10.88 ^b^	74.09 ± 1.89 ^d^	131.38 ± 4.19 ^c^

Control refer to the serum pectin from untreated tomato juice; Break-65, Break-90, US-Break-22 and US-Break-65 refer to thethermal break at 65 °C for 10 min, thermal break at 90 °C for 10 min, ultrasound break at 22 °C for 10 min, and ultrasound break at 65 °C for 10 min, respectively. GalA, Fuc, Rha, Gal, Xyl, Ara and Man refer to galacturonic acid, fucose, rhamnose, galactose, xylose, arabinose, mannose, respectively; The linearity of pectin molecules is defined by the ratio of (GalA)/(Fuc + Rha + Ara + Gal + Xyl); The length of side chain attached to RG-I is defined as (Ara + Gal)/Rha; The *DM* refer to the degree of methoxylation; The *M_w_* refer to the weight-average molecular weight. ^a–e^ Data bearing different superscript lowercase letters in the same raw are significantly different (*p* < 0.0.5).

**Table 2:** Effects of different breaking treatments on the phenolic content (µg/g DW) of the tomato pastes.

Compound	Control	Break-65	Break-90	US-Break-22	US-Break-65
rutin	185.22 ± 15.1 ^c^	196.35 ± 10.86 ^bc^	204.01 ± 10.18 ^b^	221.38 ± 14.86 ^a^	209.41 ± 10.77 ^ab^
quercetin	18.99 ± 0.80 ^b^	20.75 ± 0.90 ^b^	25.63 ± 0.45 ^a^	27.64 ± 1.78 ^a^	26.37 ± 2.23b ^a^
naringenin	79.43 ± 3.03 ^c^	95.77 ± 9.01 ^b^	111.93 ± 7.57 ^a^	114.41 ± 4.15 ^a^	99.85 ± 7.01 ^b^
naringenin-7-*O*-glucoside	22.08 ± 0.69 ^a^	21.17 ± 1.22 ^a^	22.73 ± 0.88 ^a^	24.05 ± 2.77 ^a^	23.87 ± 0.80 ^a^
total flavonols/flavanone	305.72 ± 13.72 ^d^	331.39 ± 10.17 ^c^	366.29 ± 12.52 ^b^	393.54 ± 20.43 ^a^	361.67 ± 16.47 ^b^
protocatechuic acid	2.33 ± 0.13 ^d^	3.04 ± 0.08 ^c^	2.57 ± 0.14 ^d^	4.22 ± 0.12 ^a^	3.89 ± 0.27 ^b^
ferulic acid	11.03 ± 0.06 ^d^	13.87 ± 1.08 ^b^	12.11 ± 0.23 ^c^	15.74 ± 0.54 ^a^	14.55 ± 0.36 ^b^
caffeic acid	13.76 ± 0.91 ^c^	16.9 ± 0.62 ^b^	14.74 ± 0.71 ^c^	17.91 ± 0.50 ^a^	18.53 ± 1.29 ^a^
*p*-coumaric acid	13.94 ± 1.25 ^b^	14.88 ± 0.63 ^b^	13.41 ± 0.22 ^b^	16.87 ± 0.25 ^a^	16.71 ± 1.03 ^a^
gentistic acid	16.92 ± 0.24 ^b^	20.26 ± 0.52 ^a^	17.61 ± 1.30 ^b^	20.41 ± 1.85 ^a^	20.82 ± 2.10 ^a^
chlorogenic acid	17.22 ± 1.88 ^c^	19.41 ± 1.18 ^bc^	17.58 ± 0.92 ^c^	25.52 ± 2.13 ^a^	20.75 ± 1.46 ^b^
total phenolic acid	76.48 ± 1.18 ^d^	88.37 ± 0.89 ^c^	78.01 ± 1.20 ^d^	100.69 ± 0.52 ^a^	94.88 ± 0.47 ^b^
total phenolic	382.20 ± 13.25 ^d^	419.76 ± 10.80 ^c^	444.30 ± 13.61 ^c^	494.23 ± 20.27 ^a^	456.55 ± 16.58 ^b^

Control refers to the untreated raw tomato juice; Break-65, Break-90, US-Break-22 and US-Break-65 refer to the thermal break at 65 °C for 10 min, thermal break at 90 °C for 10 min, ultrasound break at 22 °C for 10 min, and ultrasound break at 65 °C for 10 min, respectively. ^a–d^ Data bearing in different superscript lowercase letters in the same row are significantly different (*p* < 0.05).

**Table 3:** Effects of different breaking treatments on the content (mg/100 g DW) of carotenoids in tomato pastes.

Compound	Control	Break-65	Break-90	US-Break-22	US-Break-65
*cis*-lutein/ *cis*-lutein-5,8-epoxides	0.21 ± 0.01 ^ab^	0.16 ± 0.01 ^bc^	0.15 ± 0.01 ^c^	0.22 ± 0.02 ^a^	0.18 ± 0.02 ^b^
all-*trans*-lutein	4.07 ± 0.15 ^a^	3.36 ± 0.13 ^b^	3.30 ± 0.44 ^b^	3.51 ± 0.31 ^b^	4.15 ± 0.15 ^a^
13-*cis*-lutein	0.22 ± 0.02 ^b^	0.34 ± 0.04 ^a^	0.32 ± 0.04 ^a^	0.38 ± 0.04 ^a^	0.33 ± 0.02 ^a^
total lutein	4.50 ± 0.16 ^ab^	3.86 ± 0.12 ^c^	3.77 ± 0.45 ^c^	4.11 ± 0.29 ^bc^	4.67 ± 0.18 ^a^
15-*cis*-β-carotene	1.41 ± 0.14 ^c^	1.87 ± 0.08 ^b^	1.84 ± 0.14 ^b^	2.12 ± 0.03 ^a^	1.87 ± 0.16 ^b^
di-*cis*-β-carotene	1.55 ± 0.09 ^c^	1.84 ± 0.09 ^b^	1.89 ± 0.14 ^b^	2.02 ± 0.16 ^ab^	2.13 ± 0.09 ^a^
all-*trans*-β-carotene	18.08 ± 1.71 ^b^	18.06 ± 0.92 ^b^	18.00 ± 1.65 ^b^	20.76 ± 0.86 ^a^	20.35 ± 0.89 ^ab^
13-*cis*-β-carotene	0.85 ± 0.07 ^b^	1.10 ± 0.03 ^b^	1.45 ± 0.21 ^a^	1.43 ± 0.20 ^a^	1.38 ± 0.14 ^a^
total β-carotene	21.90 ± 1.57 ^b^	22.87 ± 0.99 ^b^	23.18 ± 1.45 ^b^	26.33 ± 0.94 ^a^	25.73 ± 0.84 ^a^
15-*cis*-lycopene	1.71 ± 0.08 ^b^	1.93 ± 0.11 ^b^	1.99 ± 0.17 ^b^	2.36 ± 0.19 ^ab^	2.47 ± 0.18 ^a^
13-*cis*-lycopene	4.25 ± 0.18 ^d^	4.70 ± 0.17 ^cd^	5.16 ± 0.20 ^bc^	5.41 ± 0.23 ^ab^	5.96 ± 0.58 ^a^
9,13-*di*-*cis*-lycopene	1.03 ± 0.11 ^d^	1.32 ± 0.08 ^c^	1.61 ± 0.22 ^b^	1.68 ± 0.06 ^ab^	1.87 ± 0.14 ^a^
9-*cis*-lycopene	1.88 ± 0.01 ^b^	2.23 ± 0.13 ^b^	2.73 ± 0.25 ^a^	2.79 ± 0.13 ^a^	2.65 ± 0.20 ^a^
9′-*cis*-lycopene	0.32 ± 0.03 ^a^	0.29 ± 0.00 ^a^	0.29 ± 0.00 ^a^	0.29 ± 0.01 ^a^	0.30 ± 0.03 ^a^
5,9-*cis*-lycopene	1.20 ± 0.18 ^c^	1.34 ± 0.05 ^bc^	1.54 ± 0.05 ^b^	1.92 ± 0.06 ^a^	1.94 ± 0.11 ^a^
5-*cis*-lycopene	3.13 ± 0.16 ^c^	3.64 ± 0.24 ^b^	4.29 ± 0.26 ^a^	4.22 ± 0.31 ^a^	4.19 ± 0.11 ^a^
5′-*cis*-lycopene	0.34 ± 0.02 ^b^	0.39 ± 0.02 ^b^	0.38 ± 0.03 ^b^	0.47 ± 0.04 ^a^	0.46 ± 0.03 ^a^
all-*trans*-lycopene	109.89 ± 2.91 ^a^	95.52 ± 5.91 ^bc^	87.34 ± 4.63 ^c^	103.56 ± 6.85 ^ab^	103.14 ± 11.62 ^ab^
total lycopene	123.74 ± 2.78 ^a^	111.36 ± 6.08 ^b^	105.33 ± 5.12 ^b^	122.69 ± 6.93 ^a^	122.98 ± 11.89 ^a^
total carotenoids	150.13 ± 4.19 ^a^	138.08 ± 5.28 ^b^	132.28 ± 6.89 ^b^	153.13 ± 5.92 ^a^	153.38 ± 11.4 ^a^

Control refers to the untreated raw tomato juice; Break-65, Break-90, US-Break-22 and US-Break-65 refer to the thermal break at 65 °C for 10 min, thermal break at 90 °C for 10 min, ultrasound break at 22 °C for 10 min, and ultrasound break at 65 °C for 10 min, respectively. ^a–d^ Data bearing in different superscript lowercase letters in the same row are significantly different (*p* < 0.05).

**Table 4:** Effects of different breaking treatments on the hydrophilic (HAA) and lipophilic (LAA) antioxidant activity of tomato pastes evaluated by DPPH and ABTS analysis.

		Control	Break-65	Break-90	US-Break-22	US-Break-65
ABTS (µmol TE/100g DW)	H ^d^	2279.7 ± 188.5 ^ab^	1938.3 ± 170.5 ^bc^	1812.7 ± 134.2 ^c^	2464.8 ± 276.2 ^a^	1998.1 ± 169.5 ^bc^
	L ^e^	1010.1 ± 158.2 ^c^	1325.9 ± 276.1 ^b^	1500.2 ± 138.6 ^ab^	1742.9 ± 86.4 ^a^	1798.8 ± 113.3 ^a^
	T ^f^	3289.8 ± 312.4 ^c^	3264.2 ± 233.4 ^c^	3313.0 ± 20.5 ^c^	4207.6 ± 279.5 ^b^	3796.9 ± 273.5 ^a^
DPPH (µmol TE/100g DW)	H	932.7 ± 81.5 ^b^	787.8 ± 102.3 ^c^	698.3 ± 79.3 ^c^	1247.2 ± 47.6 ^a^	823.8 ± 78.5 ^b^
	L	539.7 ± 74.6 ^c^	705.2 ± 85.0 ^b^	832.4 ± 73.8 ^b^	955.2 ± 132.1 ^a^	1037.2 ± 118.2 ^a^
	T	1472.3 ± 83.4 ^c^	1493.2 ± 154.3 ^c^	1530.7 ± 152.5 ^c^	2202.4 ± 26.0 ^a^	1861.0 ± 86.9 ^a^

Control refers to the untreated raw tomato juice; Break-65, Break-90, US-Break-22 and US-Break-65 refer to the thermal break at 65 °C for 10 min, thermal break at 90 °C for 10 min, ultrasound break at 22 °C for 10 min, and ultrasound break at 65 °C for 10 min, respectively. ^a–c^ Data bearing in different superscript lowercase letters in the same row are significantly different (*p* < 0.05). ^d^ H refers to the hydrophilic fraction. ^e^ L refers to the lipophilic fraction. ^f^ T refers to the total antioxidant activity.

## Data Availability

The data presented in this study are available on request from the corresponding author.

## Supplementary Materials

The following are available online at https://www.mdpi.com/article/10.3390/foods10102395/s1, Figure S1: Effects of different breaking treatments on the elastic modulus (*G*′) and viscous modulus (*G*″) of tomato paste as a function of strain amplitude. Figure S2: HPLC chromatogram of the standard phenolics mixture. Figure S3: Effects of different breaking treatments on the activity of PME and PG in tomato juice before concentration processing. Figure S4: Relative intensity of the third harmonic *I_3/1_* as a function of strain amplitude. Table S1: Effects of different breaking treatments on the rheological parameters of tomato paste. Table S2: Pearson’s correlation analysis of viscosity, rheological properties, particle size and the properties of the serum pectin from four different tomato pastes. Table S3: Effects of different breaking treatments on the content of carotenoids in tomato juices before concentration processing.
