# Supplementary material for: Effects of Breaking Methods on the Viscosity, Rheological Properties and Nutritional Value of Tomato Paste
Source: Foods. 2021 Oct 9;10(10):2395. doi: 10.3390/foods10102395 (PMC8535101; doi:10.3390/foods10102395)
Supplement: Supplementary file 1 [file foods-10-02395-s001.zip › Supplementary materials.pdf]

Figure captions

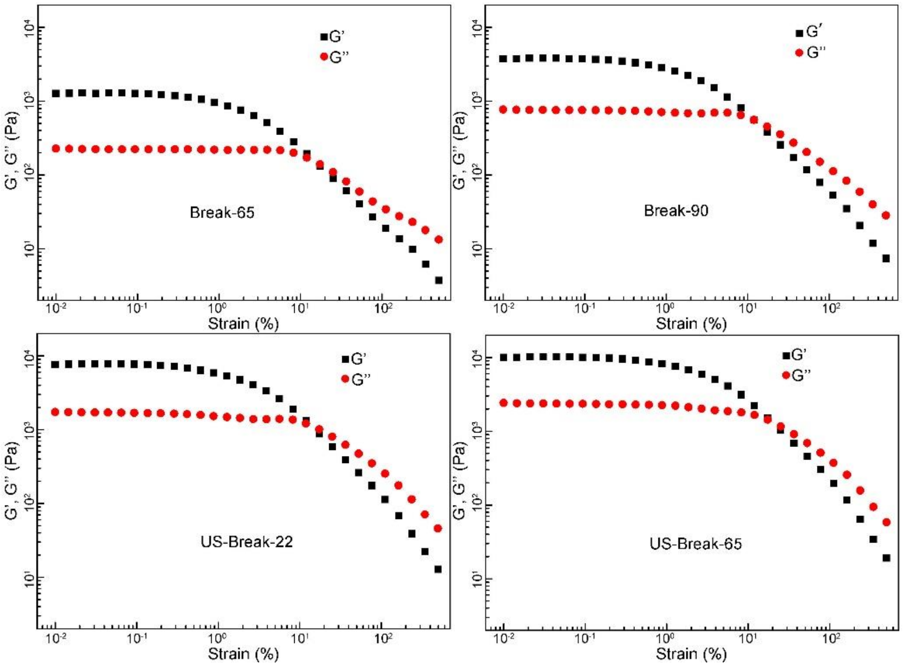

Figure S1 Effects of different breaking treatments on the elastic modulus (*G′*) and viscous modulus (*G″*) of tomato paste as a function of strain amplitude. Break-65, Break-90, US-Break-22 and US-Break-65 refer to the thermal break at 65 °C for 10 min, thermal break at 90 °C for 10 min, ultrasound break at 22 °C for 10 min, and ultrasound break at 65 °C for 10 min, respectively.

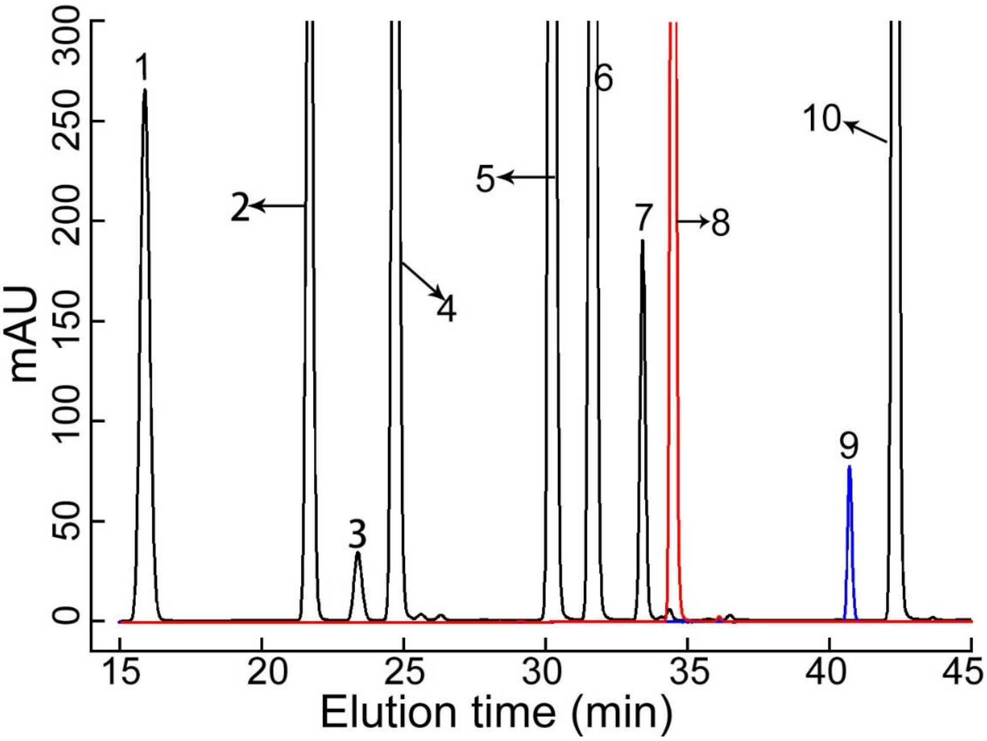

Figure S2 HPLC chromatogram of the standard phenolics mixture. Peaks 1-10 refer to protocatechuic acid, chlorogenic acid, gentistic acid, caffeic acid, *p*-coumaric acid, ferulic acid, rutin, naringenin-7-*O*-glucoside, quercetin, naringenin, respectively.

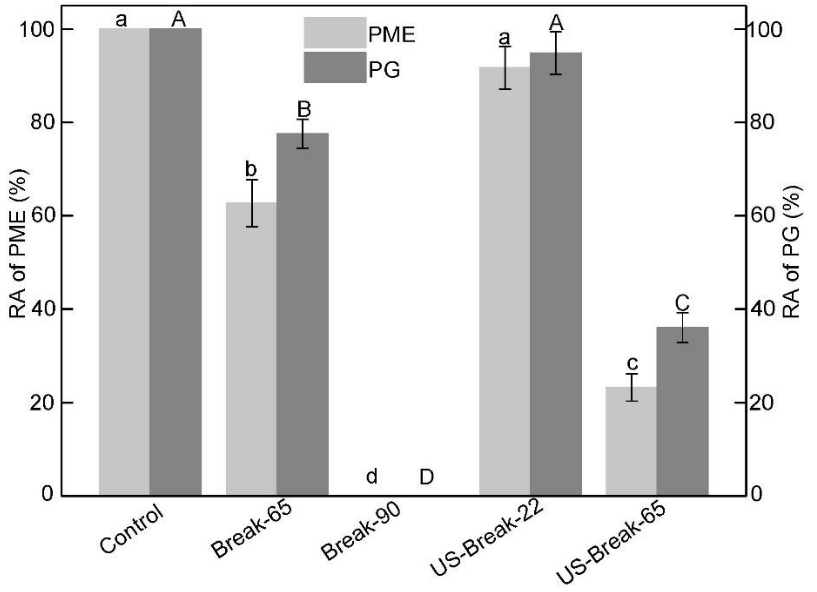

Figure S3 Effects of different breaking treatments on the activity of PME and PG in tomato juice before concentration processing. RA refer to the residual activities of the enzymes. Control refer to the untreated raw tomato juice. Break-65, Break-90, US-Break-22 and US-Break-65 refer to the thermal break at 65 °C for 10 min, thermal break at 90 °C for 10 min, ultrasound break at 22 °C for 10 min, and ultrasound break at 65 °C for 10 min, respectively. [a-d] The RA of the PME data bearing different lowercase letters were significantly different ($p < 0.05$). [A-D] The RA of PG data bearing different capital letters were significantly different ($p < 0.05$).

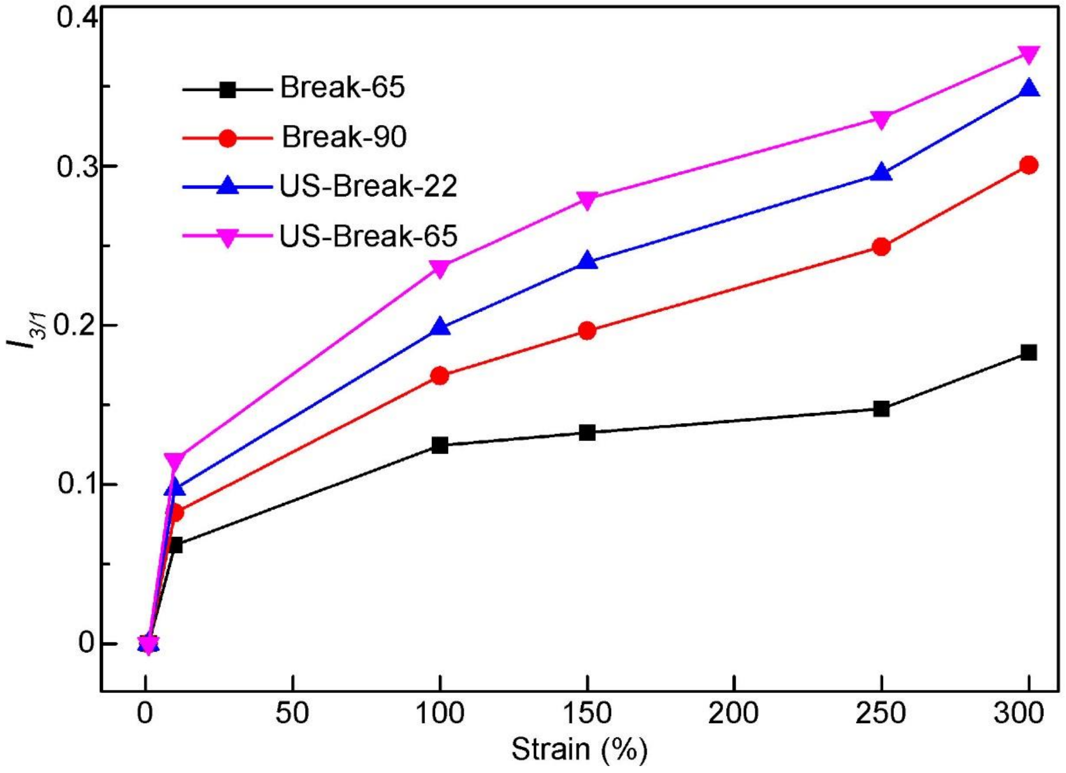

Figure S4 Relative intensity of the third harmonic $I_{3/1}$ as a function of strain amplitude. Break-65, Break-90, US-Break-22 and US-Break-65 refer to the thermal break at 65 °C for 10 min, thermal break at 90 °C for 10 min, ultrasound break at 22 °C for 10 min, and ultrasound break at 65 °C for 10 min, respectively.
