# Supplementary material for: Effects of Breaking Methods on the Viscosity, Rheological Properties and Nutritional Value of Tomato Paste
Source: Foods. 2021 Oct 9;10(10):2395. doi: 10.3390/foods10102395 (PMC8535101; doi:10.3390/foods10102395)
Supplement: Supplementary file 1 [file foods-10-02395-s001.zip › Table S1.pdf]

Table S1 Effects of different breaking treatments on the rheological parameters of tomato paste.

Parameter [e]	Break-65	Break-90	US-Break-22	US-Break-65
Data of steady shear measurements fitting to $\tau=\tau_0+K\dot{\gamma}$.				
τ_0 (Pa)	33.50 ± 5.08^{d}	58.31 ± 7.16^{c}	85.41 ± 7.16^{b}	110.72 ± 10.24^{a}
K (Pa·s^n)	39.25 ± 3.33^{d}	70.70 ± 9.23^{c}	152.30 ± 17.71^{b}	194.00 ± 12.58^{a}
n	0.50 ± 0.02^{a}	0.45 ± 0.01^{b}	0.34 ± 0.02^{c}	0.27 ± 0.03^{d}
R^2	0.99	0.99	0.99	0.99
Data of dynamic oscillatory shear measurements fitting to $G'=k'\omega^{n'}$, $G'=k''\omega^{n''}$, respectively.				
k'	$1060.61+124.74^{d}$	$2563.55+388.36^{c}$	$4368.68+281.58^{b}$	$6355.94+87.34^{a}$
n'	$0.11+0.01^{c}$	$0.13+0.01^{b}$	$0.14+0.01^{ab}$	$0.15+0.01^{a}$
R^2	0.98	0.99	0.99	0.99
k''	$105.23+14.18^{d}$	$390.12+47.29^{c}$	$648.61+46.03^{b}$	$1062.95+87.27^{a}$
n''	$0.35+0.02^{a}$	$0.28+0.01^{b}$	$0.28+0.01^{b}$	$0.27+0.01^{b}$
R^2	0.99	0.99	0.99	0.99

Break-65, Break-90, US-Break-22 and US-Break-65 refer to the thermal break at 65 °C for 10 min, thermal break at 90 °C for 10 min, ultrasound break at 22 °C for 10 min, and ultrasound break at 65 °C for 10 min, respectively. [a-d] Data bearing in different superscript lowercase letters in the same row are significantly different ($p < 0.05$). [e] Rheological parameters of steady shear include yield stress (τ_0), consistency coefficient (K), flow behavior index (n), which were obtained by fitting the shear stress (τ) and shear rate ($\dot{\gamma}$) data from the steady shear rheological curves across the specific fitting range to Herschel-Bulkley model. The rheological parameters of dynamic oscillatory shear measurements include k' and k'' refer to the consistency coefficients (Pa.s^n), while n', n'' are the behavior index.
