# Supplementary material for: Effects of Breaking Methods on the Viscosity, Rheological Properties and Nutritional Value of Tomato Paste
Source: Foods. 2021 Oct 9;10(10):2395. doi: 10.3390/foods10102395 (PMC8535101; doi:10.3390/foods10102395)
Supplement: Supplementary file 1 [file foods-10-02395-s001.zip › Table S2.pdf]

**Table S2** Pearson's correlation analysis of viscosity, rheological properties, particle size and the properties of the serum pectin from four different tomato pastes.

| | Viscosity | $\tau_0$ | $K$ | $n$ | $k'$ | $k''$ | $D_{[3,2]}$ | $D_{[4,3]}$ | Content of pectin in serum | $DM$ | $M_W$ | Linearity | Side chain length |
|---|---|---|---|---|---|---|---|---|---|---|---|---|---|
| Viscosity | 1.000 | | | | | | | | | | | | |
| $\tau_0$ | 0.993** | 1.000 | | | | | | | | | | | |
| $K$ | 0.963* | 0.988* | 1.000 | | | | | | | | | | |
| $n$ | -0.971* | -0.993** | -0.999** | 1.000 | | | | | | | | | |
| $k'$ | 0.991** | 0.998** | 0.988* | -0.993** | 1.000 | | | | | | | | |
| $k''$ | 0.993** | 0.993** | 0.974* | -0.983* | 0.997** | 1.000 | | | | | | | |
| $D_{[3,2]}$ | -0.975* | -0.978* | -0.964* | 0.974* | -0.988* | -0.994** | 1.000 | | | | | | |
| $D_{[4,3]}$ | -0.920 | -0.947 | -0.962* | 0.967* | -0.961* | -0.959* | 0.978* | 1.000 | | | | | |
| Content of pectin in serum | 0.994** | 0.983* | 0.948 | -0.955* | 0.975* | 0.975* | -0.946 | -0.877 | 1.000 | | | | |
| $DM$ | 0.162 | 0.041 | -0.111 | 0.077 | 0.034 | 0.087 | -0.054 | 0.146 | 0.201 | 1.000 | | | |
| $M_W$ | 0.398 | 0.283 | 0.135 | -0.169 | 0.278 | 0.329 | -0.297 | -0.099 | 0.430 | 0.969* | 1.000 | | |
| Linearity | 0.141 | 0.022 | -0.129 | 0.098 | 0.008 | 0.055 | -0.010 | 0.194 | 0.191 | 0.993** | 0.954* | 1.000 | |
| Side chain length | -0.016 | -0.137 | -0.286 | 0.254 | -0.146 | -0.094 | 0.128 | 0.322 | 0.027 | 0.983* | 0.909 | 0.984* | 1.000 |

Rheological parameters of steady shear include $\tau_0$, $K$ and $n$ refer to the yield stress (Pa), consistency coefficient (Pa·s$^n$), flow behavior index, respectively. The rheological parameters of dynamic oscillatory shear measurements include $k'$ and $k''$ refer to the initial storage modulus and loss modulus, respectively. $D_{[4,3]}$ and $D_{[3,2]}$ represent the volume-based mean diameter and area-based mean diameter, respectively. The $DM$ refer to the degree of methoxylation. The $M_w$ refer to the weight-average molecular weight. The linearity of pectin molecules is defined by the ratio of (GalA)/(Fuc+Rha+Ara+Gal+Xyl); The length of side chain attached to RG-I is defined as (Ara+Gal)/Rha. * and ** refer to the significant levels at 0.05 and 0.01, respectively.
