# Supplementary material for: Effects of Breaking Methods on the Viscosity, Rheological Properties and Nutritional Value of Tomato Paste
Source: Foods. 2021 Oct 9;10(10):2395. doi: 10.3390/foods10102395 (PMC8535101; doi:10.3390/foods10102395)
Supplement: Supplementary file 1 [file foods-10-02395-s001.zip › Tables S3.pdf]

Table S3 Effects of different breaking treatments on the content (mg/100 g DW) of carotenoids in tomato juices before concentration processing.

Compound	Control	Break-65	Break-90	US-Break-22	US-Break-65
cis-lutein/ *cis*-lutein-5,8-epoxides	0.21±0.01^{c}	0.22±0.02^{bc}	0.23±0.02^{bc}	0.27±0.02^{a}	0.24±0.02^{ab}
all-*trans*-lutein	4.07±0.15^{c}	4.15±0.28^{bc}	4.54±0.27^{ab}	4.96±0.23^{a}	4.72±0.14^{a}
13-*cis*-lutein	0.22±0.02^{e}	0.25±0.01^{bc}	0.28±0.03^{ab}	0.31±0.02^{a}	0.29±0.03^{ab}
total lutein	4.50±0.16^{c}	4.63±0.25^{c}	5.05±0.31^{b}	5.54±0.23^{a}	5.25±0.14^{ab}
15-*cis*-β-carotene	1.41±0.14^{c}	1.61±0.10^{bc}	1.73±0.14^{ab}	1.92±0.05^{b}	1.77±0.10^{abc}
di-*cis*-β-carotene	1.55±0.09^{b}	1.62±0.23^{ab}	1.79±0.18^{ab}	1.85±0.06^{a}	1.87±0.10^{a}
all-*trans*-β-carotene	18.08±1.71^{c}	19.75±0.61^{bc}	22.45±2.22^{b}	26.12±0.67^{a}	25.76±2.13^{a}
13-*cis*-β-carotene	0.85±0.07^{c}	0.90±0.04^{c}	1.08±0.12^{b}	1.28±0.13^{a}	1.20±0.09^{ab}
total β-carotene	21.90±1.57^{c}	23.89±0.76^{c}	27.05±2.18^{b}	31.17±0.58^{a}	30.61±2.09^{a}
15-*cis*-lycopene	1.71±0.08^{b}	1.77±0.02^{b}	1.86±0.09^{b}	2.24±0.18^{a}	2.11±0.13^{a}
13-*cis*-lycopene	4.25±0.18^{c}	4.51±0.20^{bc}	4.84±0.57^{ab}	5.20±0.17^{a}	5.05±0.06^{ab}
9,13-*di-cis*-lycopene	1.03±0.11^{c}	1.17±0.08^{a}	1.39±0.10^{c}	1.64±0.21^{ab}	1.54±0.06^{b}
9-*cis*-lycopene	1.88±0.01^{c}	2.03±0.21^{bc}	2.41±0.22^{a}	2.67±0.27^{a}	2.33±0.11^{ab}
9'-*cis*-lycopene	0.32±0.03^{a}	0.35±0.04^{a}	0.25±0.01^{b}	0.27±0.01^{b}	0.27±0.03^{b}
5,9-*cis*-lycopene	1.20±0.18^{c}	1.23±0.02^{c}	1.45±0.14^{b}	1.89±0.06^{a}	1.73±0.11^{a}
5-*cis*-lycopene	3.13±0.16^{c}	3.48±0.07^{bc}	3.65±0.24^{b}	4.11±0.22^{a}	3.89±0.32^{ab}
5'-*cis*-lycopene	0.34±0.02^{c}	0.37±0.03^{c}	0.38±0.04^{bc}	0.45±0.02^{a}	0.43±0.02^{ab}
all-*trans*-lycopene	109.89±2.91^{c}	115.20±8.75^{b}	119.53±6.14^{b}	128.55±3.46^{a}	121.42±3.41^{ab}
total lycopene	123.74±2.78^{c}	130.10±8.79^{bc}	135.77±6.07^{ab}	147.03±3.57^{a}	138.76±3.68^{ab}
total carotenoids	150.13±4.19^{c}	150.26±4.4^{c}	167.87±8.38^{b}	183.74±3.11^{a}	174.62±5.53^{ab}

Control refer to the untreated raw tomato juice; Break-65, Break-90, US-Break-22, and US-Break-65 refer to the thermal break at 65 ^{o}C, thermal break at 90 ^{o}C, ultrasound break at 22 ^{o}C and ultrasound break at 65 ^{o}C, respectively. $^{a\text{-}c}$ Data bearing in different superscript lowercase letters in the same row are significantly different ($p < 0.05$).
